# Vaccine Knowledge, Awareness and Hesitancy: A Cross Sectional Survey among Parents Residing at Sandakan District, Sabah

**DOI:** 10.3390/vaccines9111348

**Published:** 2021-11-17

**Authors:** James Yau Hon Voo, Qi Ying Lean, Long Chiau Ming, Nur Hafzan Md. Hanafiah, Yaser Mohammed Al-Worafi, Baharudin Ibrahim

**Affiliations:** 1 School of Pharmaceutical Science, Universiti Sains Malaysia, Gelugor 11800, Malaysia; jamesvooyh@moh.gov.my (J.Y.H.V.); hafzanhanafiah@usm.my (N.H.M.H.); 2 Faculty of Pharmacy, Universiti Teknologi MARA, Cawangan Pulau Pinang, Kampus Bertam, Kepala Batas, Pulau Pinang 13200, Malaysia; leanqiying@yahoo.com; 3 PAP Rashidah Sa’adatul Bolkiah Institute of Health Sciences, Universiti Brunei Darussalam, Gadong, Bandar Seri Begawan BE1410, Brunei; longchiauming@gmail.com; 4 College of Medical Sciences, Azal University for Human Development, Amran P.O. Box 447, Yemen; yworafi@yahoo.com; 5 College of Pharmacy, University of Science and Technology of Fujairah, Fujairah P.O. Box 2202, United Arab Emirates; 6 Faculty of Pharmacy, Universiti Malaya, Kuala Lumpur 50603, Malaysia

**Keywords:** vaccine hesitancy, immunization, questionnaire, vaccination

## Abstract

Background: Incomplete childhood immunization against communicable diseases is a major concern and vaccine hesitancy remains a hurdle to overcome in primary vaccination programs. This study was to examine the parents’ vaccine knowledge, awareness and hesitancy in relation to their children’s immunization status. Methods: A cross-sectional questionnaire study design was used. The parents who brought their children for immunization visit or follow-up at four public health clinics located in Sandakan district were invited to participate in this survey. Informed consent was obtained before each participant completed a hard copy of self-administered questionnaire in either English or Malay versions. Results: Of 405 parents responded, they generally had good knowledge and awareness of vaccines, only a small percentage (6.8%) of parents were found vaccine hesitant. There were significant differences in vaccine knowledge and awareness in those from different education levels and employment status; similarly, these two factors also significantly affected the vaccine hesitancy among the parents. The parents’ knowledge score was found to be moderately associated with their awareness (r = 0.551, *p* < 0.01) and inversely correlated to vaccine hesitancy (r = −0.397, *p* < 0.01). Most of the children (*n* = 376, 92.8%) in the study were immunized. The children’s immunization status was significantly associated with the parents’ education level (*p* = 0.025). There was also a significant difference in the total vaccine knowledge scores between the groups of parents with different child immunization status (*p* = 0.05). Conclusion: This study revealed that parents with higher education had a better knowledge of vaccinations, were less vaccine hesitant and were more likely to ensure that their children complete the recommended course of immunization. It is crucial to ensure parents are well-informed about the safety and efficacy of vaccines so that the children are protected from communicable diseases by the child vaccination program.

## 1. Introduction

Vaccination is one of the most cost-effective public health interventions and has substantially reduced the mortality and morbidity of children from vaccine-preventable diseases, including invasive pneumococcal diseases, diphtheria-tetanus-pertussis (DTaP), measles, mumps, rubella, polio, and *Haemophilus influenzae* type b (Hib) [1]. The combination of immunization and other healthcare development interventions has reduced the annual number of deaths among children under five years of age from approximately 9.6 million in 200 to 5.2 million in 2019, a decline of more than 46% [2]. By rolling out childhood immunizations in the 73 poorest countries in the world over the next decade, it is estimated that treatment cost and lost wages can be reduced by $1.4 billion and $313 million, respectively. In addition, $61 billion of the long-term costs associated with loss of productivity due to disability and death could be saved [2].

In Malaysia, more than 90% of children receive Bacillus Calmette–Guérin (BCG), Hepatitis B, DTaP, Polio (IPV) and Hib vaccines up to the three recommended doses [3]. Despite the extensive national immunization program and the high vaccination rates, some vaccine-preventable diseases remain a challenge in Malaysia. For instance, the incidence of measles increased by nearly six-fold from 0.73 per 1000 population in 2014 to 4.32 per 1000 population in 2015 [3]. Similarly, in the year 2014 to 2015, there was a two-fold increase in the incidence of both neonatal tetanus and pertussis from 0.02 to 0.05 per 1000 population and 1.65 to 3.08 per 1000 population, respectively [3]. According to the Malaysia National Health and Morbidity Survey (NHMS) 2016, there has been an increasing prevalence of incomplete immunization and non-immunization in Malaysia with the rate approaching 5% [4,5].

The reemergence of the aforementioned vaccine-preventable diseases reflects inappropriate immunization coverage in non-immunized children. Several studies have shown that the immunization status of children is highly dependent on the knowledge and awareness of the parents [6,7]. Parents with good knowledge were found to have better understanding regarding the role of vaccine in protecting their children from various illnesses [7]. Nevertheless, there were also group of parents with false impression about vaccines due to lack of knowledge and incorrect information on vaccine schedule [8]. Some concerned parents also noted that they are not aware of reliable information regarding vaccination from the local, regional and national media [9]. They mainly obtain the information of immunization from other parents with vaccinated children [10]. In addition, the Strategic Advisory Group of Experts on Immunization (SAGE) Working Group on Vaccine Hesitancy showed that the immunization status of children could be affected by the hesitancy of the parents [11,12]. Numerous comprehensive studies have been conducted in the Western and other Asian countries to evaluate the knowledge, awareness, and hesitancy of parents regarding childhood immunization. A few studies conducted by Western researchers reported that around 9–28% of parents were hesitant to let their children receive vaccinations in accordance with the immunization schedule [13,14,15]. The high vaccine hesitancy among parents has led to decreased immunization coverage among the children.

Similarly, it is not uncommon to see reports on parental refusal to vaccination in Malaysia [16,17]. Nonetheless, the concerns of the refusal group have not been successfully addressed by public health vaccination awareness programs [17]. A few factors including vaccine-related issues, parental health, cultural and religious beliefs were identified barriers against vaccination for children from West Malaysia [18]. As ethnicity could be an underlying influential factor of vaccine acceptance, it is important to gauge the knowledge, awareness and vaccine hesitancy in the population of East Malaysia, which represents approximately one fifth of the population of Malaysia. Sabah is the second largest state in Malaysia, comprising people of various ethnicities, which are widely spread in different areas. Sabah has 32 ethnic groups with the majority Kadazan Dusun being Sabah Bumiputera (indigenous peoples) [19]. We aimed to investigate how sociodemographic factors were associated with vaccine knowledge, awareness and hesitancy among parents in East Malaysia. We also aimed to identify how sociodemographic factors, knowledge and awareness were associated with actual vaccination coverage among children.

## 2. Materials and Methods

### 2.1. Study Design and Subjects

This was a cross-sectional study that assessed parental vaccine knowledge, vaccine awareness and vaccine hesitancy based on a self-administered questionnaire from February to March 2018. Vaccine knowledge is defined as detailed and factual information regarding vaccines and immunization whereas awareness is defined as having personally relevant and adequate information regarding vaccines and immunization [6]. Vaccine hesitancy refers to the delay in acceptance or refusal despite availability of vaccination services [11]. Parents who brought their children (between 0–4 years) for an immunization visit or follow-up at four public health clinics in Sandakan district were eligible under the inclusion criteria. However, parents whose children were immunocompromised (e.g., Human Immunodeficiency Virus positive, malignancy) were excluded from this study. The sample size for a survey was calculated using Raosoft sample size calculator (www.raosoft.com). The number of respondents was based on the reported number of children aged from 0–4 years old in year 2010 in Sandakan, with the population of 33,088. With a 95% confidence interval and 5% margin of error, the calculated sample size was 380. As convenient sampling was used to recruit the respondents, in view of possibility of about 20% dropout or incompleteness, a total 450 parents were invited to participate in this study. Informed consent was obtained from each participant before they were self-administered the questionnaire and advised to seek clarification from the investigators if there were any queries. The questionnaires were checked when they were handed in to ensure completeness.

The study was conducted in compliance with ethical principles outlined in the Declaration of Helsinki and Malaysian Good Clinical Practice Guideline 4th edition. In addition, an ethical approval was obtained from the Medical Research Ethics Committee (MREC) and Clinical Research Center (CRC) for this study (NMRR-17-2912-38792).

### 2.2. The Data Collection Instrument

The set of pre-validated questionnaire, with permission, was adapted from two previous studies [6,13]. The original English version of the questionnaire was translated to local Malay language by two language teachers and two field experts who are competent in both languages to ensure content and conceptual equivalence. The Malay version of questionnaire was translated back to English to compare to the original texts for harmonization. The Malay version was then pilot tested in a small group of parents, all feedback were used for finalization of the questionnaire. The final questionnaire (Supplementary Material) had five sections with a total of 46 items, namely part 1: parents’ socio-demographic data (10 items), part 2: (a) vaccine knowledge (10 items), (b) vaccine awareness (10 items), (c) vaccine hesitancy (15 items), and (d) the child’s immunization schedule record (1 item). Part 2a of the questionnaire evaluated the vaccine knowledge of the respondents, with each correct answer being allocated one (1) point and each wrong answer or item with no response (don’t know) being given zero (0) point. Part 2b focused on the vaccine awareness, with each ‘yes’ and ‘no’ answer being given one (1) and zero (0) point, respectively. The scores of total vaccine knowledge and total vaccine awareness of the parents were, respectively, derived by adding up the scores for each question in Part 2a and Part 2b, where the minimum score was 0 and the maximum score was 10. Participants’ overall knowledge and awareness score was, respectively, categorized using Bloom’s cut-off point; good for a score between 8 and 10 points, moderate for a score between 6 and 7.9 points, and poor for a score of less than 6 points [20]. Vaccine hesitancy was assessed in Part 2c which consisted of three domains: behavior (Item 1 and 2); safety and efficacy (Item 7–10); and general attitudes (Item 3–6 and Item 11–15). The scoring of the questions was determined by giving two (2) points for each statement leading to the vaccine hesitancy; one (1) point for the option ‘don’t know’; and zero (0) point for the option indicating the acceptance of vaccination. The item scores were totaled up to a total score ranging from 0 to 30. Subsequently, the total hesitancy score was then converted to percentage, which ranged from 0 to 100. A score of ≥50 points indicated hesitancy whereas score <50 was non-hesitant [13]. Lastly, the median for the vaccine knowledge, vaccine awareness, and vaccine hesitancy scores were all calculated. Additionally, the immunization status of the respondents’ children was determined based on the children’s immunization schedule booklet. If there were no record for the administration on due dates, the children’s immunization status was considered incomplete and grouped as not up to date.

### 2.3. Statistical Analysis

The data collected were tabulated and analyzed using the Statistical Package for Social Sciences (SPSS) version 16.0. Descriptive statistics were used to show the distribution of parents’ sociodemographic characteristics and their children’s immunization status. In addition, the total vaccine knowledge, total vaccine awareness and total vaccine hesitancy scores were calculated.

Statistical analysis was performed on all demographic parameters for comparison, with the *p*-value < 0.05 being considered as statistically significant. Due to the non-parametric distribution of the scores obtained, Mann-Whitney U and Kruskal-Wallis tests were employed to determine the differences in vaccine knowledge scores, vaccine awareness scores, and vaccine hesitancy scores in each sociodemographic characteristic. Furthermore, the Spearman’s correlation was used to assess the association between total vaccine knowledge scores, total vaccine awareness scores, and total vaccine hesitancy scores. The Chi-square test and Fisher exact test were conducted to evaluate the association between the parents’ sociodemographic parameters and the children’s immunization status groups (up to date versus not up-to-date). On the other hand, the Mann-Whitney U test was used to compare the total vaccine knowledge scores, total vaccine awareness scores, and total vaccine hesitancy scores between the children’s immunization status groups (up to date versus not up-to-date)

## 3. Results

### 3.1. Sociodemographic Characteristics in Relation to Vaccine Knowledge, Awareness and Hesitancy

A total of 450 questionnaires were distributed of which, 405 questionnaires were completed and returned, giving rise to a response rate of 90%. The sociodemographic characteristics of parents are summarized in Table 1. The majority of respondents were mothers (95.8%) with a mean age of 30.4 years. About two-thirds of them had 1–2 children, and three quarters (75.6%) of them were living in urban areas with an education level of at least secondary school or higher. Only about 40% of the participants were employed and had a family monthly income of more than Malaysian ringgit 2000 (USD 465).

### 3.2. Vaccine-Related Scores in Relation to Parents’ Sociodemographic Characteristics

For total vaccine knowledge and vaccine awareness scores, the median (IQR) was 7.0 (5.0–8.0) and 8.0 (6.0–10.0), respectively. Most parents had good (score ≥ 8) awareness about the vaccines although their knowledge was generally moderate to good (score ≥ 6). On the other hand, the median (IQR) of the total vaccine hesitancy score was 16.67 (10.0–30.0), indicating mostly parents were non-hesitant (score < 50%) about their children’s vaccination.

Table 1 also details the total vaccine knowledge score, total vaccine awareness score and total vaccine hesitancy score differences in relation to the parents’ sociodemographic characteristics. The median vaccine knowledge scores were higher among older parents, those living in the urban areas, employed, with a secondary school or higher education level, and with a family income of more than MYR2000 compared to younger parents, those from rural areas, unemployed or with lower income and or education. Interestingly, other than living in the urban areas, employed, with a secondary school or higher education level, and with a family income of more than MYR2000, parents who had less than five children were also found to achieve higher vaccine awareness scores. Likewise, parents who were unemployed and less educated (secondary school level or lower) were shown to have higher vaccine hesitancy scores.

### 3.3. Vaccine Hesitancy by Sociodemographic Characteristics

Of total respondents, only 27 (6.8%) out of the 405 parents were vaccine-hesitant (score ≥ 50%) (Table 2). The vaccine hesitancy was significantly associated with the parents’ employment status (*p* = 0.018) and education level (*p* = 0.08).

### 3.4. The Relationships between Vaccine-Related Scores

We then investigated the relationships between vaccine knowledge, awareness and vaccine hesitancy using the Spearman correlation analysis. There was a significant moderate positive correlation between the parents’ vaccine knowledge and their vaccine awareness (r = 0.551, *p* < 0.01). The parents’ vaccine hesitancy was negatively correlated with vaccine knowledge (r = −0.397, *p* < 0.01) and vaccine awareness (r = −0.351, *p* < 0.01), respectively.

### 3.5. Children’s Immunization Status and Its Relationship with Parents’ Sociodemographic Characteristics and Vaccine-Related Scores

In this study, the majority of the children (*n* = 376, 92.8%) were immunized according to the immunization schedules (Table 3). The children’s immunization status was significantly associated with parental education level (*p* = 0.025). The higher the level of the parents’ education level, the more of their children had up-to-date immunization. The other sociodemographic characteristics of the parents were not significantly associated with the children’s immunization status.

### 3.6. Relationship of Vaccine-Related Scores Difference by the Children’s Immunization Status Groups

There was a significant difference (*p* = 0.05) in the parents’ total vaccine knowledge scores between the two groups with different children’s immunization status (Table 4). However, no significant differences were observed for the parents’ total vaccine awareness and hesitancy scores between the two groups (*p* > 0.05).

## 4. Discussion

This study aimed to investigate how sociodemographic factors were associated with vaccine knowledge, awareness and hesitancy among parents in East Malaysia. The findings of this study indicate that most parents, especially mothers, had good knowledge and awareness of vaccines. Only a small percentage (6.8%) of them were vaccine hesitant, and 7.2% of their children did not complete their vaccination on time. There was a significant difference of children’s immunization status among parents with different education levels. The parents’ vaccine knowledge, awareness and vaccine hesitancy were affected by the education levels and employment status.

In previous studies, there were a higher proportion of parents having vaccine hesitancy and more children had not been fully vaccinated [13]. For example, in the study of vaccine hesitancy among parents in a tertiary hospital in Kuala Lumpur, 11.6% of parents were found to be vaccine hesitant. On the other hand, a study investigating childhood immunization status among children aged less than two years in public maternal child health clinics in the Tawau district in Sabah showed that 24.8% had incomplete immunization status [21]. In another district of Sabah, Kota Kinabalu recorded 16.8% of defaulting immunization among children aged 12 to 24 months in all government maternal child health clinics [13]. Previous studies had revealed certain factors including low awareness or poor knowledge about the benefits of vaccination and the use of alternative medicines especially among mothers as being associated with vaccine hesitancy in Malaysia [13,18,22]. In the current study, mothers (95.8% of the parents) might be the persons affecting the immunization status of their children. Some individuals perceive that vaccination is unnecessary and may increase the risk of falling sick after immunization and believe that the infectious diseases no longer exist in the community [18]. Others have religious concerns or beliefs, are unsure about the effectiveness of vaccines, or have concerns about the safety and side effects of vaccines [23]. In the US, safety-related issues being the main reason for vaccine hesitancy, up to one-third of respondents opted not to acquire vaccines in surveys on COVID-19 vaccine acceptance [24].

In this study, the socioeconomic background of parents affected vaccine knowledge, awareness, and hesitancy scores. Similarly, numerous previous studies concluded that the parents’ vaccine knowledge, awareness and hesitancy were associated their education level [25,26,27]. The parents who had completed secondary school and tertiary education achieved higher vaccine knowledge and awareness scores and lower hesitancy scores [28]. Well- educated parents tend to process the evidence available regarding vaccination and understand the information better [29]. Similarly, parents with higher education background are usually working parents. Their better knowledge and awareness about vaccinations could be a result of information seeking and sharing among the colleagues who were also parents [26]. In addition, the employed parents might perceive vaccination positively, and therefore be less vaccine-hesitant compared to the unemployed parents [13,30]. In contrast, the parents who had no or primary education and were unemployed might have less knowledge, lack exposure to accurate information about vaccination or rely more on the social media or word-of-mouth, as highlighted in a recent study [31]. These could lead to misleading or wrong interpretation regarding immunization, thus resulting in increased misconception and vaccine hesitancy among parents [32,33]. In agreement with previous studies, we also found a significant association between the parents’ education level and the children’s immunization status [34]. The children whose parents are more educated tend to have a complete immunization status.

Furthermore, the findings of this study are supported by several studies which found that the completeness of the children’s immunization was not associated with the parental age, ethnicity, place of living, employment status, and family income [34]. Even though we had hypothesized that ethnicity or religion may influence decisions on childhood immunization, our findings revealed that the immunization status in children is not affected by the ethnicities and religions. These could be explained by immunization services being widely provided by easily accessible healthcare facilities in Malaysia in both urban and rural areas. Furthermore, the costs of the standard children vaccinations are fully covered by the government, making the services available and free of charge to all Malaysian children regardless of the parents’ socioeconomic status. Nonetheless, this research established a significant difference in the parents’ vaccine knowledge scores between the children’s immunization status groups, which was consistent with many other previous studies [13,35,36]. It can be inferred from these findings that parents having a better knowledge about vaccinations have a higher probability of their children being vaccinated. It is believed that the interventions to raise awareness of childhood vaccination uptake are just as important as the endeavors to enhance the parental vaccine knowledge in increasing the rate of childhood immunization [37].

Surprisingly, we did not observe significant association between hesitancy score and immunization. One would expect that parents who are hesitant towards vaccines, they would tend to refuse vaccination in their children. However, vaccine hesitancy does not necessarily translate into vaccine refusal. Some are adamant refusers but with appropriate information and education by healthcare providers, others may be convinced to vaccinate their children.

There are links between educational attainment, employment status and family income. It was not unexpected that there were significant differences in the parents’ vaccine knowledge and awareness scores between groups with different family income and place of living. The parents with greater income were found to have better vaccine knowledge and awareness. This finding was consistent with many other studies conducted in Asia where the parental knowledge was directly proportional to the family income [26,27]. This could be explained by wealthy parents tending to be tech-savvy and have access to more healthcare resources, including paying for optional vaccines such as Rotavirus, Hepatitis A vaccines which are not included in the national child immunization program. In addition, giving people more options of vaccines might increase vaccination acceptance and uptake yet meeting each individual vaccine preferences is not feasible during emerging pandemic such as COVID-19 [24]. In terms of vaccine hesitancy, there was no significant difference in the total scores in different family income groups, which was consistent with a local study [13]. Nevertheless, some found that the low income among the unemployed parents could be a contributing factor for increasing vaccine hesitancy [38]. Consistent with several previous studies, we found that the parents living in the urban areas had better vaccine knowledge and awareness compared to those from in the rural areas [6,39,40]. A plausible explanation would be the tendency of the parents living in the urban areas to have higher education and thus higher income than those in the rural areas. In addition, the parents in the urban areas have better access to healthcare facilities, including private clinics and community pharmacies, where information about vaccinations is available.

In this study, there was a difference in vaccine knowledge between parents with different age groups, but this was not observed for their scores in vaccine awareness and hesitancy. Many previous studies show that older parents have better immunization knowledge than the younger parents [6,25,26,41]. As for vaccine hesitancy, parental age was not a related factor, but other studies seem to suggest otherwise [13,32,42]. According to previous studies, younger parents were more vaccine-hesitant than the older parents as they were less experienced and had less knowledge about the vaccine side effects and vaccine- preventable diseases. In addition, younger parents relied more on social media for information about vaccinations which may not always provide sufficient or accurate information [13,32,42]. Interestingly, a small but significant difference of parents’ vaccine awareness between the different family sizes was also observed in this study. Parents with less than five children demonstrated higher vaccine awareness scores as compared to the parents with more than five children. This is in contrast to a study from China, where parents with more children had greater vaccine awareness score [43]. Our result could possibly be explained by parents in bigger families being too busy to juggle between work and family, and therefore failing to take their children for vaccination. However, among the groups, we did not observe any difference in vaccine knowledge and vaccine hesitancy while other studies have shown that parents with more than three children have better knowledge about immunization and lower vaccine hesitancy than the parents with 2 or less children [13,42].

Our study has several limitations. Firstly, this study involved the parents, mainly mothers (95.8% of the respondents) living in Sandakan, Sabah. Therefore, the findings may not be representative of the vaccine knowledge, vaccine awareness, vaccine hesitancy and immunization status in other parts of East Malaysia, including different districts of Sabah and the state of Sarawak. Future studies could include both parents of the two East Malaysian states of Sabah and Sarawak in rural and urban health clinics. Secondly, this study was based on a survey; hence its findings should be interpreted carefully and used only as a baseline to determine the factors influencing the children’s immunizations status. Qualitative studies may also be useful in order to explore more complex issues regarding the factors affecting vaccine knowledge, vaccine awareness and vaccine hesitancy. Lastly, this study could be carried out in the community setting, not only limited to public healthcare facilities to give a better depiction of the immunization acceptance and status in Malaysia.

## 5. Conclusions

Parents who have good knowledge and awareness of vaccines are less hesitant to vaccines. There remain a small number of parents (6.8%) who were vaccine hesitant and up to 7.2% of children had not completed their vaccination in this study. Vaccine knowledge, awareness, and hesitancy among parents were affected by various sociodemographic characteristics, including the education level, employment status, place of living, family income, age and family size. Furthermore, the children’s immunization status was significantly associated with the parents’ education level, children whose parents had a higher education were more likely to be fully vaccinated. Correspondingly, parental knowledge about vaccinations was associated with the completeness of the children’s immunization, suggesting that educational interventions could effectively improve the vaccination rates. Future studies or educational interventions should focus on improving parent’s knowledge, in the aim of a higher vaccine acceptance and vaccination coverage in children. All stakeholders including policymakers, healthcare providers, and non-government organizations must emphasize good communication with parents in order to ensure that their children are vaccinated.

## Figures and Tables

**Table 1:** Total Vaccine Knowledge, Awareness, and Hesitancy Score by Parents’ Sociodemographic Characteristics.

Sociodemographic	Frequency (%)	Knowledge Scores Median (IQR)	*p*-Value	Awareness Scores Median (IQR)	*p*-Value	Hesitancy Scores (%) Median (IQR)	*p*-Value
Gender *			0.619		0.885		0.150
Female	388 (95.8)	7.0 (5.0–8.0)		8.0 (6.0–10.0)		16.67 (10.0–30.0)	
Male	17 (4.2)	7.0 (5.5–7.5)		8.0 (6.5–10.0)		23.33 (13.33–30.0)	
Age (year-old) **			**0.03** **2**		0.595		0.706
18–20	15 (3.7)	6.0 (5.0–8.0)		7.0 (6.0–10.0)		20.0 (3.3–33.3)	
21–25	64 (15.8)	6.0 (4.0–7.8)		8.0 (5.0–9.0)		23.3 (10.0–39.2)	
26–30	138 (34.1)	7.0 (5.0–8.0)		8.0 (6.0–10.0)		16.67 (10.0–30.0)	
31–35	122 (30.1)	7.0 (5.0–8.0)		8.0 (6.0–10.0)		16.67 (6.7–30.0)	
36–40	49 (12.1)	7.0 (5.0–9.0)		9.0 (6.0–10.0)		16.67 (10.0–26.7)	
>40	17 (4.2)	8.0 (7.0–9.0)		8.0 (7.0–9.5)		16.67 (13.3–22.5)	
Marital Status *			0.191		0.886		0.516
Married	40 (98.8)	7.0 (5.0–8.0)		8.0 (6.0–10.0)		16.7 (10.0–30.0)	
Others	5 (1.2)	8.0 (6.5–8.5)		9.0 (6.0–9.5)		18.3 (4.2–20.0)	
Number of children **			0.929		**0.04** **2**		0.826
1–2	261 (64.4)	7.0 (5.0–8.0)		8.0 (6.0–10.0)		16.7 (10.0–28.3)	
3–5	126 (31.1)	7.0 (4.75–8.0)		8.0 (6.0–9.0)		16.7 (10.0–32.5)	
>5	18 (4.5)	7.0 (4.0–8.3)		7.0 (4.0–9.0)		20.0 (9.2–34.2)	
Ethnicity **			0.051		0.132		0.649
Malay	98 (24.2)	7.0 (5.0–8.0)		9.0 (6.0–10.0)		20.0 (13.3–26.7)	
Chinese	15 (3.7)	8.0 (7.0–8.0)		9.0 (6.0–10.0)		16.67 (6.7–23.3)	
Indian	2 (0.5)	6.0 (5.0–7.0)		5.0 (4.0–6.0)		23.33 (16.7–36.7)	
Sabah Bumiputera	290 (71.6)	7.0 (5.0–8.0)		8.0 (6.0–10.0)		16.67 (6.7–33.3)	
Religion *			0.453		0.431		0.072
Muslim	362 (89.4)	7.0 (5.0–8.0)		8.0 (6.0–10.0)		16.67 (10.0–26.7)	
Other religions	43 (10.6)	7.0 (6.0–8.0)		8.0 (6.0–10.0)		20.0 (13.3–40.8)	
Place of living *			**0.01** **6**		**<0.01**		0.493
Rural	99 (24.4)	6.0 (4.0–8.0)		7.0 (5.0–9.0)		16.67 (10.0–33.3)	
Urban	306 (75.6)	7.0 (5.0–8.0)		9.0 (6.0–10.0)		16.67 (8.3–30.0)	
Employment status *			**<0.01**		**0.0** **2**		**0.03** **5**
Unemployed	245 (60.5)	6.0 (5.0–8.0)		8.0 (6.0–9.0)		20.0 (10.0–33.3)	
Employed	160 (39.5)	7.0 (6.0–8.0)		9.0 (7.0–10.0)		16.67 (6.7–26.7)	
Education level **			**<0.01**		**<0.01**		**0.0** **2**
No formal education	23 (5.7)	5.0 (3.0–7.0)		6.0 (3.0–9.0)		36.7 (11.7–48.3)	
Primary school	91 (22.5)	6.0 (4.0–8.0)		7.0 (5.0–9.0)		20.0 (10.0–30.0)	
Secondary school	225 (55.6)	7.0 (5.0–8.0)		8.0 (6.50–10.0)		16.7 (10.0–30.0)	
Tertiary education	66 (16.3)	7.0 (6.0–8.0)		9.0 (7.75–10.0)		13.3 (6.7–21.7)	
Family monthly income **			**<0.01**		**<0.01**		0.601
Less than MYR2000	246 (60.7)	6.0 (4.0–8.0)		8.0 (5.0–9.0)		18.3 (10.0–33.3)	
MYR2000–<MYR5000	127 (31.4)	7.0 (6.0–8.0)		9.0 (7.0–10.0)		16.67 (6.7–26.7)	
MYR5000–MYR10,000	21 (5.2)	8.0 (7.0–9.0)		9.0 (8.0–10.0)		16.67 (11.7–25.0)	
More than MYR10,000	11 (2.7)	7.0 (6.0–8.0)		9.0 (8.0–10.0)		26.67 (6.7–46.7)	

* Mann-Whitney U, ** Kruskal-Wallis, *p* < 0.05. Bumiputera (indigenous peoples).

**Table 2:** Association between Parents’ Sociodemographic Characteristics and vaccine hesitancy.

Sociodemographic	Group *n* (%)		Total	*p*-Value
	Non-Hesitant	Hesitant		
Gender **				0.071
Female	354 (89.7)	24 (6.1)	378 (95.8)	
Male	14 (3.5)	3 (0.7)	17 (4.2)	
Age (year-old) **				0.710
18–20	14 (3.5)	1 (0.3)	15 (3.8)	
21–25	54 (13.7)	6 (1.5)	60 (15.2)	
26–30	127 (32.2)	10 (2.5)	137 (34.7)	
31–35	113 (28.6)	6 (1.5)	119 (30.1)	
36–40	44 (11.1)	4 (1.0)	48 (12.1)	
>40	16 (4.1)	0 (0.0)	16 (4.1)	
Marital Status **				0.753
Married	364 (92.2)	27 (6.8)	391 (99.0)	
Others	4 (1.0)	0 (0.0)	4 (1.0)	
Number of children **				0.759
1–2	236 (59.8)	17 (4.3)	253 (64.1)	
3–5	116 (29.4)	8 (2.0)	124 (31.4)	
>5	16 (4.0)	2 (0.5)	18 (4.5)	
Ethnicity **				0.881
Malay	90 (22.8)	5 (1.3)	95 (24.1)	
Chinese	14 (3.5)	1 (0.3)	15 (3.8)	
Indian	2 (0.5)	0 (0.0)	2 (0.5)	
Sabah Bumiputera	262 (66.3)	21 (5.3)	283 (71.6)	
Religion **				0.465
Muslim	330 (83.6)	23 (5.8)	353 (89.4)	
Other religion	38 (9.6)	4 (1.0)	42 (10.6)	
Place of living *				0.548
Rural	90 (22.8)	8 (2.0)	98 (24.8)	
Urban	278 (70.4)	19 (4.8	297 (75.2)	
Employment status *				**0.018**
Unemployed	215 (54.4)	22 (5.6)	237 (60.0)	
Employed	153 (38.7)	5 (1.3)	158 (40.0)	
Education level **				**0.08**
No formal education	16 (4.0)	5 (1.3)	21 (5.3)	
Primary school	81 (20.5)	8 (2.0)	89 (22.5)	
Secondary school	209 (52.9)	11 (2.8)	220 (55.7)	
Tertiary education	62 (15.7)	3 (0.8)	65 (16.5)	
Family monthly income **				0.118
Less than MYR2000	218 (55.2)	20 (5.1)	238 (60.3)	
MYR2000–<MYR5000	121 (30.6)	4 (1.0)	125 (31.6)	
MYR5000–MYR10,000	20 (5.0)	1 (0.3)	21 (5.3)	
More than MYR10,000	9 (2.3)	2 (0.5)	11 (2.8)	

* Chi-Square, ** Fisher-exact, *p* < 0.05. Bumiputera (indigenous peoples).

**Table 3:** Association between Parents’ Socio-demographic Characteristics and Children’s Immunization Status.

Sociodemographic	Group *n* (%)		Total	*p*-Value
	Up-to-Date	Not Up-to-Date		
Gender **				0.348
Female	361 (93.0)	27 (7.0)	388 (95.8)	
Male	15 (88.2)	2 (11.8)	17 (4.2)	
Age (year-old) **				0.429
18–20	14 (93.3)	1 (6.7)	15 (3.7)	
21–25	57 (89.1)	7 (10.9)	64 (15.8)	
26–30	132 (95.7)	6 (4.3)	138 (34.1)	
31–35	111 (91.0)	11 (9.0)	122 (30.1)	
36–40	47 (95.9)	2 (4.1)	49 (12.1)	
>40	15 (88.2)	2 (11.8)	17 (4.2)	
Marital Status **				0.312
Married	372 (93.0)	28 (7.0)	40 (98.8)	
Others	4 (80.0)	1 (20.0)	5 (1.2)	
Number of children **				0.226
1–2	245 (93.9)	16 (6.1)	261 (64.4)	
3–5	116 (92.0)	10 (8.0)	126 (31.1)	
>5	15 (83.3)	3 (16.7)	18 (4.4)	
Ethnicity **				0.983
Malay	91 (92.9)	7 (7.1)	98 (24.2)	
Chinese	14 (93.3)	1 (6.7)	15 (3.7)	
Indian	2 (10)	0 (0.0)	2 (0.5)	
Sabah Bumiputera	269 (66.4)	21 (5.2)	290 (71.6)	
Religion **				1.00
Muslim	336 (92.8)	26 (7.2)	362 (89.4)	
Other religions	40 (93.0)	3 (7.0)	43 (10.6)	
Place of living *				0.968
Rural	92 (92.9)	7 (7.1)	99 (24.4)	
Urban	284 (92.8)	22 (7.2)	306 (75.6)	
Employment status *				0.173
Unemployed	224 (91.4)	21 (8.6)	245 (60.5)	
Employed	152 (95.0)	8 (5.0)	160 (39.5)	
Education level **				**0.025**
No formal education	19 (82.6)	4 (17.4)	23 (5.7)	
Primary school	83 (91.2)	8 (8.8)	91 (22.5)	
Secondary school	210 (93.3)	15 (6.7)	225 (55.6)	
Tertiary education	64 (97.0)	2 (3.0)	66 (16.3)	
Family monthly income **				0.775
Less than MYR2000	226 (91.9)	20 (8.1)	246 (60.7)	
MYR2000–<MYR5000	120 (94.5)	7 (5.5)	127 (31.4)	
MYR5000–MYR10,000	20 (95.2)	1 (4.8)	21 (5.2)	
More than MYR10,000	10 (90.9)	1 (9.1)	11 (2.7)	

* Chi-Square, ** Fisher-exact, *p* <0.05. Bumiputera (indigenous peoples).

**Table 4:** Total Vaccine Knowledge, Awareness and Hesitancy Score Differences between Children’s Immunization Status Groups.

Parameter	*n* (%)	Knowledge Scores Median (IQR)	*p*-Value	Awareness Scores Median (IQR)	*p*-Value	Hesitancy Scores Median (IQR)	*p*-Value
Immunization status *			**0.05**		0.980		0.231
Up to Date	376 (92.8)	7.0 (5.0–8.0)		8.0 (6.0–10.0)		16.67 (10.0–30.0)	
Not Up to Date	29 (7.2)	6.0 (3.0–7.0)		8.0 (7.0–10.0)		20.0 (10.0–45.0)	

* Mann-Whitney U, *p* < 0.05.

## Data Availability

All data have been presented in this manuscript.

## Supplementary Materials

The following are available online at https://www.mdpi.com/article/10.3390/vaccines9111348/s1. File S1: The final questionnaire.
