# Supplementary material for: Vaccine Knowledge, Awareness and Hesitancy: A Cross Sectional Survey among Parents Residing at Sandakan District, Sabah
Source: Vaccines (Basel). 2021 Nov 17;9(11):1348. doi: 10.3390/vaccines9111348 (PMC8624080; doi:10.3390/vaccines9111348)
Supplement: Supplementary file 1 [file vaccines-09-01348-s001.zip › vaccines-1325231-supplementary.pdf]

Supplementary material

Questionnaire

PART 1 Parent's socio-demographic

Please tick (/) in the appropriate box

Gender	☐ Male	☐ Female		
Age	_____			
Marital status	☐ *Married*	☐ *Divorced*	☐ *Widow/Widower*	
Number of children	_____			
Race	☐ Malay	☐ *Chinese*	☐ *Indian*	☐ *Others (______________)*
Religion	☐ *Islam*	☐ *Buddhism*	☐ *Christianity*	☐ *Others (____________)*
Place of living	☐ *Rural*	☐ *Urban*		
Employment Status	☐ *Employed*	☐ *Unemployed*		
Educational level	☐ *No formal education*	☐ *Primary school*		
	☐ *Secondary school*	☐ *Tertiary education*		
Family income	☐ *Less than RM2000*	☐ *RM2000 – RM5000*		
	☐ *RM5000 – RM10000*	☐ *More than RM10000*		

Please answer all the following:

PART 2(a)

<u>Please tick (/) in the appropriate box.</u>

No.	Questions	Yes	No	Don't Know
1	*Healthy children do not need immunization.*			
2	*There are different types of vaccines.*			
3	*Active immunization is a killed or weakened form of a disease-causing agent.*			
4	*Vaccination is for all ages.*			
5	*Children get too many vaccines in the first two years of life.*			
6	*The immunization of the children should be started at birth.*			
7	*In some health situations, vaccines should not be given.*			
8	*Vaccines can be given in combination.*			
9	*If the child receives extra immunization, it is more effective and safer.*			
10	*More than one dose of vaccine may be required for complete protection.*			

PART 2(b)

<u>Please tick (/) in the appropriate box.</u>

No.	Questions	Yes	No
1	*Were you informed about vaccination?*		
2	*Did you read about vaccination in the media?*		
3	*Did you see a television programme about vaccination?*		
4	*Did you hear about vaccination on the radio?*		
5	*Did you read about vaccination on the internet?*		
6	*Did you obtain information about vaccination from an antenatal clinic?*		
7	*Did you obtain information about vaccination from a maternity hospital or home?*		
8	*Do you think you have access to enough information on immunization?*		
9	*Do you know your child's immunization appointment?*		
10	*Is it important to follow immunization schedule?*		

PART 2(c)

Please tick (/) in the appropriate box.

No.	*Questions*	*Yes*	*No*	*Don't Know*
1	*Have you ever delayed having your child get a shot for reasons other than illness or allergy?*			
2	*Have you ever decided not to have your child get a shot for reasons other than illness or allergy?*			

3	*How sure are you that following the recommended shot schedule is a good idea for your child?*										
Not at all sure											*Completely sure*
	0	1	2	3	4	5	6	7	8	9	10

4	*Children get more shots than are good for them.*				
	Strongly disagree	*Disagree*	*Not sure*	*Agree*	*Strongly agree*
5	*I believe that many of the illnesses that shots prevent are severe.*				
	Strongly disagree	*Disagree*	*Not sure*	*Agree*	*Strongly agree*
6	*It is better for my child to develop immunity by getting sick than to get a shot.*				
	Strongly disagree	*Disagree*	*Not sure*	*Agree*	*Strongly agree*
7	*It is better for children to get fewer vaccines at the same time.*				
	Strongly disagree	*Disagree*	*Not sure*	*Agree*	*Strongly agree*
8	*How concerned are you that your child might have a serious side effect from a shot?*				
	Very concerned	*Somewhat concerned*	*Not sure*	*Not too concerned*	*Not concerned at all*
9	*How concerned are you that any one of the childhood shots might not be safe?*				
	Very concerned	*Somewhat concerned*	*Not sure*	*Not too concerned*	*Not concerned at all*

10	*How concerned are you that a shot might not prevent the disease?*				
	Very concerned	*Somewhat concerned*	*Not sure*	*Not too concerned*	*Not concerned at all*

	Question	*Yes*	*No*	*Don't Know*
11	*If you had another infant today, would you want him/her to get all the recommended shots?*			

12	*Overall, how hesitant about childhood shots would you consider yourself to be?*				
	Very hesitant	*Somewhat hesitant*	*Not sure*	*Not too hesitant*	*Not hesitant at all*

13	*I trust the information I receive about shots.*				
	Strongly disagree	*Disagree*	*Not sure*	*Agree*	*Strongly agree*
14	*I am able to openly discuss my concerns about shots with my child's doctor.*				
	Strongly disagree	*Disagree*	*Not sure*	*Agree*	*Strongly agree*

15	*All things considered, how much do you trust your child's doctor?*										
Not at all sure											*Completely sure*
	0	1	2	3	4	5	6	7	8	9	10

PART 2(d) Child's immunization schedule record
Date of birth:
Gender:

Age	*Vaccine*	*Date of vaccination*
Birth	BCG	
	Hepatitis B (1)	
1 *month*	Hepatitis B (2)	
2 *months*	DPT (1)	
	Hib (1)	
	IPV (1)	
3 *months*	DPT (2)	
	Hib (2)	
	IPV (2)	
5 *months*	DPT (3)	
	Hib (3)	
	IPV (3)	
6 *months*	Hepatitis B (3)	
	Measles	
9 *months*	MMR (1)	
12 *months*	MMR (2)	
18 *months*	DPT (B)	
	Hib (B)	
	IPV (B)	

Date of data collection: ___________________
